# Angiographic biomarkers are significant predictors of treatment response to intravitreal aflibercept in diabetic macular edema

**DOI:** 10.1038/s41598-023-35286-2

**Published:** 2023-05-19

**Authors:** Martin Hein, Aleksandar Vukmirovic, Ian J. Constable, Vignesh Raja, Arman Athwal, K. Bailey Freund, Chandrakumar Balaratnasingam

**Affiliations:** 1 Lions Eye Institute, Perth, Australia; 2 Centre for Ophthalmology and Visual Science, University of Western Australia, Perth, Australia; 3 Department of Ophthalmology, Sir Charles Gairdner Hospital, Perth, Australia; 4 Joondalup Eye Clinic, Perth, Australia; 5 School of Engineering Science, Simon Fraser University, Burnaby, BC Canada; 6 Department of Medical Physics and Biomedical Engineering, University College London, London, England; 7 Vitreous Retina Macula Consultants of New York, New York, USA; 8 Department of Ophthalmology, NYU Grossman School of Medicine, New York, NY USA

**Keywords:** Biomarkers, Outcomes research, Retinal diseases

## Abstract

This prospective single-center study aims to identify biomarkers that predict improvement in best-corrected visual acuity (BCVA) and central retinal thickness (CRT) at 6 months, in 76 eyes with diabetic macular edema (DME) treated monthly with intravitreal aflibercept. At baseline, all patients underwent standardized imaging with color photography, optical coherence tomography (OCT), fluorescein angiography (FA) and OCT angiography (OCTA). Glycosylated hemoglobin, renal function, dyslipidemia, hypertension, cardiovascular disease and smoking were recorded. Retinal images were graded in a masked fashion. Baseline imaging, systemic and demographic variables were investigated to detect associations to BCVA and CRT change post aflibercept. Predictors of BCVA improvement included greater macular vessel density quantified using OCTA (*p* = 0.001) and low-density lipoprotein (LDL) ≥ 2.6 mmol/L (*p* = 0.017). Lower macular vessel density eyes showed a significant reduction in CRT but no BCVA improvement. Predictors of CRT reduction included peripheral non-perfusion seen on ultrawide-field FA (*p* = 0.005) and LDL ≥ 2.6 mmol/L (*p* < 0.001). Retinal angiographic biomarkers derived from OCTA and ultrawide-field FA may help predict functional and anatomic response to anti-vascular endothelial growth factor (VEGF) therapy in patients with DME. Elevated LDL is associated with treatment response in DME. These results may be used to better-select patients who will benefit from intravitreal aflibercept for treatment of DME.

## Introduction

Diabetic macular edema (DME) is a leading cause of vision loss in patients with diabetes mellitus (DM) and the number of people worldwide with clinically significant DME is projected to increase from 18.8 million in 2020 to 28.6 million in 2045^1^. Upregulation of vascular endothelial growth factor (VEGF) plays a key role in the pathogenesis of DME by increasing retinal microvascular permeability^2^. Antagonizing the actions of VEGF using intravitreal anti-VEGF molecules has become an effective and, in many centers, first-line treatment for DME^2^. However, up to 40% of eyes with DME demonstrate a sub-optimal response to anti-VEGF injections^3^. Currently, treatment failure can only be reliably determined by assessing changes in best corrected visual acuity (BCVA) and central retinal thickness (CRT) after a sequence of intravitreal therapies. Biomarkers predictive of a favorable treatment response, prior to its initiation, could enable a more tailored approach to DME treatment.

Retinal imaging forms the cornerstone of DME management. Historically, 7-field Early Treatment Diabetic Retinopathy Study (ETDRS) color photography was the mainstay imaging modality for grading diabetic retinopathy (DR) severity and assessing its complications^4^. However, this technique is now frequently supplemented with optical coherence tomography (OCT), fluorescein angiography (FA) and/or OCT angiography (OCTA). The widespread adoption of ultrawide field (UWF) imaging has also permitted elucidation of critical relationships between peripheral retinal changes and the macular complications of DR. The ophthalmic literature contains many detailed studies reporting the efficacy of laser and intravitreal therapies in DME management^5–8^. A limited number of these reports have performed statistical modelling using contemporaneously acquired modern multimodal retinal imaging data to establish predictors of treatment response^9–19^. Some of these previous predictive models did not account for key systemic variables such as glycemic control, dyslipidemia and renal function that are known to modulate the natural course of DR^19–21^.

We present a prospective, longitudinal study that comprehensively investigates systemic, demographic and multimodal imaging factors that might correlate with structural and visual outcomes in patients treated with intravitreal aflibercept injection (IAI) for DME. We leverage state-of-the-art high-resolution retinal imaging to explore whether retinal angiographic biomarkers are significant predictors of visual and structural outcomes in DME. This is a real-world clinical study that includes patients that have previously received treatment with ant-VEGF (with validated wash out periods) or laser therapy. This study also includes some patients with good visual acuity that demonstrated OCT-based documentation of DME progression and were judged to be at risk of vision loss. The results of this real-world work may facilitate better selection of patients that will benefit from IAI for the management of DME.

## Results

### Baseline demographic and imaging characteristics

The study group comprised of 76 eyes from 48 patients of which 29 patients were male. The demographic and non-imaging baseline features of the cohort are summarized in Table 1. Mean age of subjects was 59.9 ± 12.8 years. 8 (16.7%) patients had Type 1 DM and mean HbA1c at baseline visit was 8.6 ± 2.0%. Half of the of eyes (51.3%) had prior treatment with retinal laser, 39.5% had prior treatment with anti-VEGF and 4.9% had prior treatment with intravitreal steroids, all greater than 6 months prior to baseline imaging. The baseline retinal imaging grading and objective OCTA analysis of the cohort are summarized in Table 2. In the subjective analysis of imaging features between the two masked graders, there was a disagreement rate of 23.3% that needed to be reconciled by the third grader. We speculate this disagreement rate is in part due to the specificity of questions during grading. For example: ‘proportion of fluorescein leakage from microaneurysms’, questions such as this are susceptible to clinical interpretation.

**Table 1:** Demographic and non-imaging baseline features.

Age (years; mean ± SD)	59.9 ± 12.8
Male, no. (%)	29 (60.4)
Duration of DM (years; mean ± SD)	20.7 ± 9.4
HbA1c (%; mean ± SD)	8.6 ± 2.0
HbA1c ≤ 8%, no. (%)	27 (56.2)
HbA1c > 8%, no. (%)	21 (43.8)
Insulin use, no. (%)	19 (39.6)
Smoking, no. (%)	8 (16.7)
eGFR (mL/min/1.73m^2^; mean ± SD)	70.8 ± 22.9
Creatinine (µmol/L; mean ± SD)	101.6 ± 78.5
LDL (mmol/L; mean ± SD)	2.5 ± 1.0
HDL (mmol/L; mean ± SD)	1.2 ± 0.4
On lipid lowering therapy, no. (%)	44 (57.9)
Hypertension, no. (%)	55 (72.4)
Myocardial infarction, no. (%)	15 (19.7)
Strokes, no. (%)	4 (5.3)
Past PRP, no. (%)	39 (51.3)
Pseudophakic, no. (%)	22 (28.9)
Previous intravitreal VEGF, no. (%)	30 (39.5)
Previous intravitreal steroids, no. (%)	3 (4.9)
Previous vitrectomy, no. (%)	3 (4.9)

*DM * Diabetes mellitus; *eGFR *  Estimated Glomerular Filtration Rate; * HbA1c * Glycated hemoglobin: * HDL*  High density lipoprotein; *LDL  * Low density lipoprotein; *PRP *  Pan-retinal photocoagulation; *SD *  Standard deviation; *V EGF * Vascular endothelial growth factor.

**Table 2:** Imaging features recorded at baseline.

Imaging features	No. (%)
Presence of exudates	34 (44.7)
**Microaneurysms**	
No microaneurysms	2 (2.6)
< 10 microaneurysms	26 (33.3)
> 10 microaneurysms	48 (63.2)
**Macula fluorescein leakage pattern**	
Focal	22 (29.3)
Intermediate	20 (26.7)
Diffuse	33 (44.0)
Non-perfusion outside ETDRS 7-field grid on UWF FA	33 (46.5)
**OCT features**	
Presence of Subretinal Fluid	17 (22.7)
Cystoid changes in the INL	51 (68.0)
Cystoid changes in the ONL	71 (95.9)
Presence of DRiL	19 (25.7)
Intact ELM and EZ	62 (83.8)
Presence of intraretinal HRF	59 (79.7)
**OCTA features**	
Intact terminal foveal capillary ring	20 (26.7)
Perifoveal capillary loss	62 (82.7)
Foveal avascular zone	–
Area, perimeter, diameter, axis ratio, eccentricity, acircularity	–
Macular vessel density	–

*DRiL*  Disorganization of Retinal inner Layers; *ELM* = External Limiting Membrane; *ETDRS *  Early Treatment Diabetic Retinopathy Study; *EZ * Ellipsoid Zone; *HRF* Hyperreflective Foci; *INL* Inner Nuclear Layer; *OCT* Optical Coherence Tomography; *OCTA * Optical Coherence Tomography Angiography; *ONL* Outer Nuclear Layer; *UWF FA *  Ultra Widefield Fluorescein Angiography.

### Predictors of visual outcomes

Mean BCVA at baseline and final visits were 71.5 (20/40) and 74.7 (20/32) ETDRS letter score, respectively. Predictor variables at baseline visit and their association with an improvement in BCVA at month 6 are summarized in Table 3. Factors that were significantly associated with better visual outcomes included higher macular VD as quantified using OCTA (*p* = 0.001, c = 120.1 ETDRS letters), LDL ≥ 2.6 mmol/L (*p* = 0.017, c = 1.90 ETDRS letters), and Type 1 DM (*p* = 0.014, c = 3.99 ETDRS letters). VD, LDL and Type 1 DM were also significantly associated with an excellent BCVA outcome of ETDRS score ≥ 70. Similarly, an intact ELM/EZ was significantly associated with an excellent BCVA (*p* = 0.034) but not a BCVA improvement (*p* = 0.05). Factors that did not appear to influence BCVA improvement are summarized in Table 3 and included prior VEGF/steroid intravitreal therapy, systemic co-morbidities, glycemic control, renal function and peripheral non-perfusion.

**Table 3:** Baseline variables and association with an improvement in BCVA, excellent BCVA and CRT reduction at 6 months in eyes with diabetic macular edema treated with monthly aflibercept.

Baseline variables	*P* Value		
	BCVA Improvement	Excellent BCVA	CRT Reduction
**Demographic features**			
Age	0.52	0.56	0.27
Sex	0.09	**0.004**	0.92
**Co-morbidities**			
Type 1 DM	**0.014**	**0.002**	0.71
Insulin use	0.48	0.57	0.81
Hypertension	0.20	0.86	0.91
Lipid lowering therapy	0.31	0.81	0.49
Smoking	0.50	0.96	0.94
Stroke	0.43	0.62	0.78
Myocardial Infarction	0.28	0.14	0.70
**Ocular history**			
Pseudophakic	0.51	0.32	0.17
Previous PRP	0.95	0.64	0.72
Previous anti-VEGF therapy	0.53	0.94	0.38
Previous intravitreal steroids	0.43	0.97	0.96
Previous vitrectomy	0.14	0.65	0.74
**Laboratory measures**			
HbA1c	0.34	0.77	0.29
eGFR	0.47	0.83	0.56
Creatinine	0.89	0.67	0.69
LDL (≥ 2.6 mmol/L)	**0.017**	**< 0.001**	**< 0.001**
HDL	0.45	0.63	0.57
**Imaging features**			
Microaneurysms	0.76	0.62	0.63
Hard exudates	0.39	0.16	0.22
Fluorescein leakage pattern	0.60	0.67	0.99
Peripheral non-perfusion	0.78	0.69	**0.005**
Presence of subretinal fluid	0.53	0.90	0.14
Cystoid changes in the INL	0.79	0.30	0.96
Cystoid changes in the ONL	0.82	0.50	0.79
Presence of DRiL	0.37	0.71	0.76
Intact ELM and EZ	**0.05**	**0.034**	0.15
Presence of intraretinal HRF	0.18	0.79	0.43
Intact terminal foveal capillary ring	0.14	0.051	0.11
Perifoveal capillary loss	0.88	0.29	0.47
FAZ: area, axis ratio, eccentricity, perimeter, acircularity, maximum/minimum diameter	> 0.55	> 0.30	> 0.18
Macular vascular density	**0.001**	**0.002**	0.75

Significant *p* values are in bold.

Excellent BCVA = ETDRS letter score ≥ 70; CRT = 1 mm Central Retinal Thickness; BCVA = Best Corrected Visual Acuity; DM = Diabetes Mellitus; DRiL = Disorganization of the Retinal inner Layers; eGFR = estimated Glomerular Filtration Rate; ELM = External Limiting Membrane; ETDRS = Early Treatment Diabetic Retinopathy Study; EZ = Ellipsoid Zone; FAZ = Foveal Avascular Zone; HbA1c = Glycated Hemoglobin; HDL = High Density Lipoprotein; HRF = Hyper-reflective Foci; INL = Inner Nuclear Layer; LDL = Low Density Lipoprotein; ONL = Outer Nuclear Layer; PRP = Pan-retinal Photocoagulation; VEGF = Vascular Endothelial Growth Factor.

**Figure 1:**
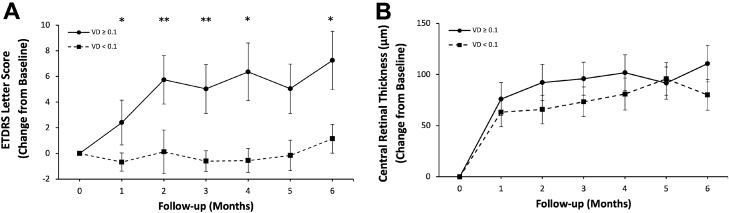
Comparisons between Early Treatment Diabetic Retinopathy Study (ETDRS) Letter Score, (**A**) and Central Retinal Thickness (CRT; µm), (**B**) change from baseline with macular vessel densities (VD) ≥ 0.1 and < 0.1 (quantified on optical coherence tomography angiography) in eyes with diabetic macular edema over six months with monthly 2 mg Aflibercept intravitreal injections. * = *p* < 0.05; ** = *p* < 0.001.

From the quantifiable OCTA factors, the mean macular VD for the cohort was 0.098 ± 0.018 (range 0.05–0.14) with a non-normally distributed negatively skewed dataset. Log transformation did not normalize the data set, however by taking a midpoint value of 0.1 (median = 0.097) the sample sizes either side of the cut-off are similar (n = 43 for VD < 0.1 and n = 32 for VD ≥ 0.1), suggesting that using this 0.1 cut-off is a fair selection for this data set. Through taking this midpoint VD value of 0.10 it was found that eyes with VD ≥ 0.10 had a greater improvement in BCVA than eyes with VD < 0.10. Baseline and final BCVA in eyes with VD ≥ 0.1 was 71.0 ± 16.3 ETDRS letters and 77.7 ± 11.5 ETDRS letters, respectively. The change in BCVA and CRT at each visit for groups with VD ≥ 0.1 and VD < 0.1 is illustrated in Fig. 1. For eyes with preserved macula VD ≥ 0.1 there was a mean increase in 6.7 ETDRS letters by 6 months (*p* = 0.015) and over the whole timeframe these eyes demonstrated a continual improvement in BCVA. The rate of improvement was seen to plateau at 4 months with the greatest increase in BCVA occurring during the first and second month when compared to eyes with VD < 0.1 at the same time points (*p* = 0.007). Eyes with VD < 0.1 did not experience a significant improvement in BCVA over any time point in the 6-month study. Baseline and final BCVA for this low macula VD group were 71.7 ± 9.2 ETDRS letters and 72.7 ± 8.8 ETDRS letters respectively. Furthermore, both the preserved macula VD and low macula VD group showed no CRT difference between each other, with no statistically significant differences in CRT between eyes with VD ≥ 0.1 (373.0 ± 118.6 µm) and eyes with VD < 0.1 at baseline (405.8 ± 127.0 µm; *p* = 0.27) and at the final visit (304.7 ± 55.7 µm vs 300.6 ± 60.2 µm; *p* = 0.790).

Patients with high LDL levels ≥ 2.6 mmol/L (100 mg/dL) experienced greater improvements in BCVA (*p* = 0.017, c = 1.90 ETDRS letters). The level of 2.6 mmol/l was chosen as the cut off value as population studies have shown that the LDL range in DM patients should optimally be below 2.6 mmol/L^22^. Patients above and below this 2.6 mmol/L cut off both demonstrated an improvement in BCVA at 6 months but those in the higher LDL group demonstrated greater improvements. Patients with LDL < 2.6 mmol/L showed an average improvement of 1.8 ETDRS letters at their final visit from baseline (71.6 ± 10.4 ETDRS letters) while patients with LDL ≥ 2.6 mmol/L showed an average improvement of 4.0 ETDRS letters at their final visit from baseline (73.7 ± 12.3 ETDRS letters).

**Figure 2:**
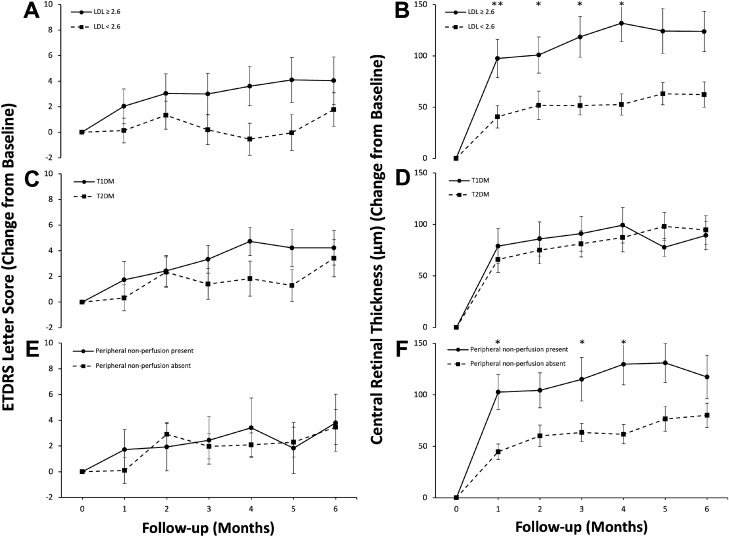
Comparisons between Early Treatment Diabetic Retinopathy Study (ETDRS) Letter Score and Central Retinal Thickness (CRT; µm) change from baseline in high (≥ 2.6 mmol/L) and low LDL (low density lipoprotein; < 2.6 mmol/L), (**A**, **B**), Type 1 Diabetes Mellitus (T1DM) and Type 2 Diabetes Mellitus (T2DM), (**C**, **D**) and those with and without peripheral non-perfusion (seen on ultrawide field fluorescein angiography), (**E**, **F**) in eyes with diabetic macular edema over six months with monthly 2 mg Aflibercept intravitreal injections. * = *p* < 0.05; ** = *p* < 0.001.

Patients with Type 1 DM had a larger BCVA improvement than Type 2 DM (Fig. 2). Though baseline BCVA of Type 1 DM and Type 2 DM were significantly different (*p* = 0.002). This significance carried through to the final visit with BCVA of Type 1 DM being greater than Type 2 DM patients.

Fifty eyes (65.8%) achieved an excellent visual outcome where BCVA was 20/40 (≥ 70 ETDRS letter score) or better at the final visit. Though 36 of these 50 eyes had ≥ 70 ETDRS letter score at baseline. Predictor variables at baseline visit and their association with excellent visual outcome at month 6 are summarized in Table 3. The factors that were significantly associated with excellent visual outcomes included VD (*p* = 0.002, c = -125.3 ETDRS letters), intact EZ/ELM (*p* = 0.034, c = -5.3 ETDRS letters), LDL (*p* < 0.001, c = 2.1 ETDRS letters), Type 1 DM (*p* = 0.002, c = 4.6 ETDRS letters) and Male Sex (*p* = 0.004, c = 3.7 ETDRS letters).

### Predictors of CRT reduction

Mean 1 mm CRT at baseline and final visits were 388.8 µm and 300.5 µm, respectively. Predictor variables at baseline visit and their association with reduction in 1 mm CRT measurements at month 6 are summarized in Table 3.

**Figure 3:**
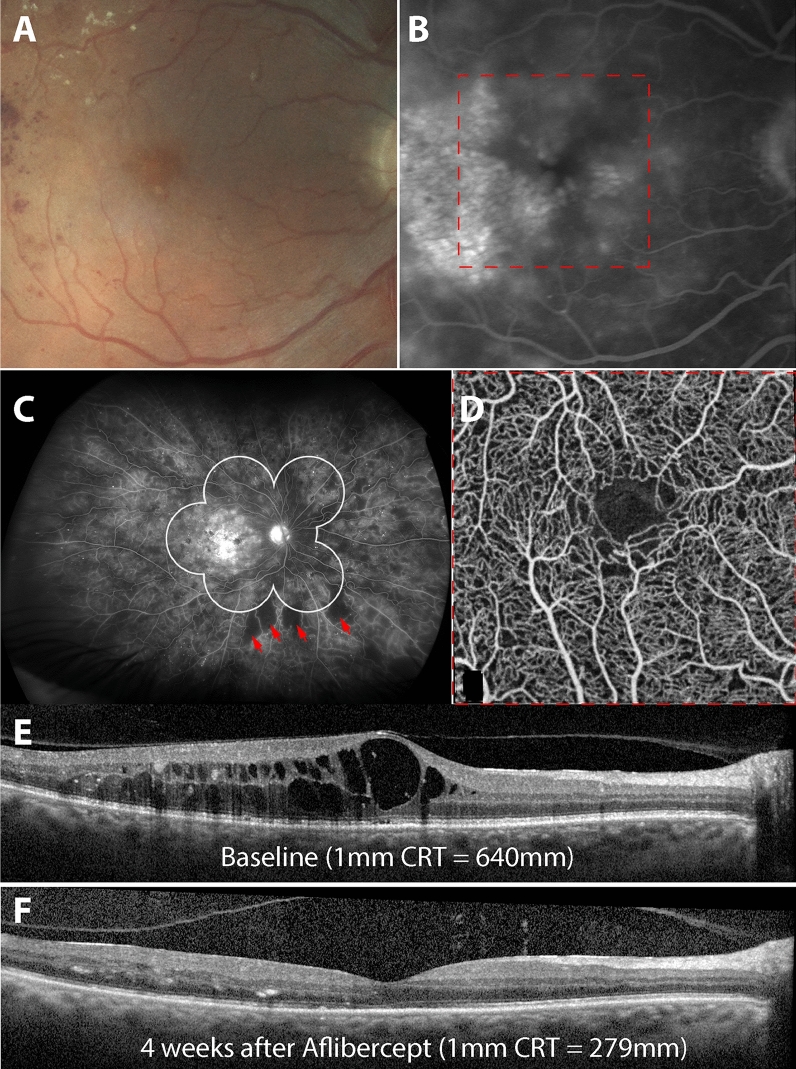
Central retinal thickness (CRT) changes in eyes with evidence of retinal non-perfusion outside the Early Treatment Diabetic Retinopathy Study (ETDRS) 7-field grid. The imaging features of a 62 year-old male with Type 2 Diabetes Mellitus are presented. (**A**) Fundus photograph (Canon CX-1). (**B**)**,** Fluorescein angiography (FA) of the macula (Heidelberg Spectralis). (**C**)**,** Ultrawide field FA (Optos California). (**D**)**,** Optical coherence tomography angiography (OCTA) of the macula (Optovue XR Avanti). (**E**), Single B-scans of the central fovea at baseline and (**F**), 4 weeks after treatment with 2 mg of aflibercept are provided (Heidelberg Spectralis). Note that there has been marked reduction in central retinal thickness (CRT) following treatment in this patient that has evidence of retinal non-perfusion outside the ETDRS 7-field grid (red arrows). The FA also demonstrates leakage in the central macula, staining of peripheral retinal vessels and leakage from the peripheral retinal circulation. Perifoveal capillary density is mostly preserved on OCTA.

**Figure 4:**
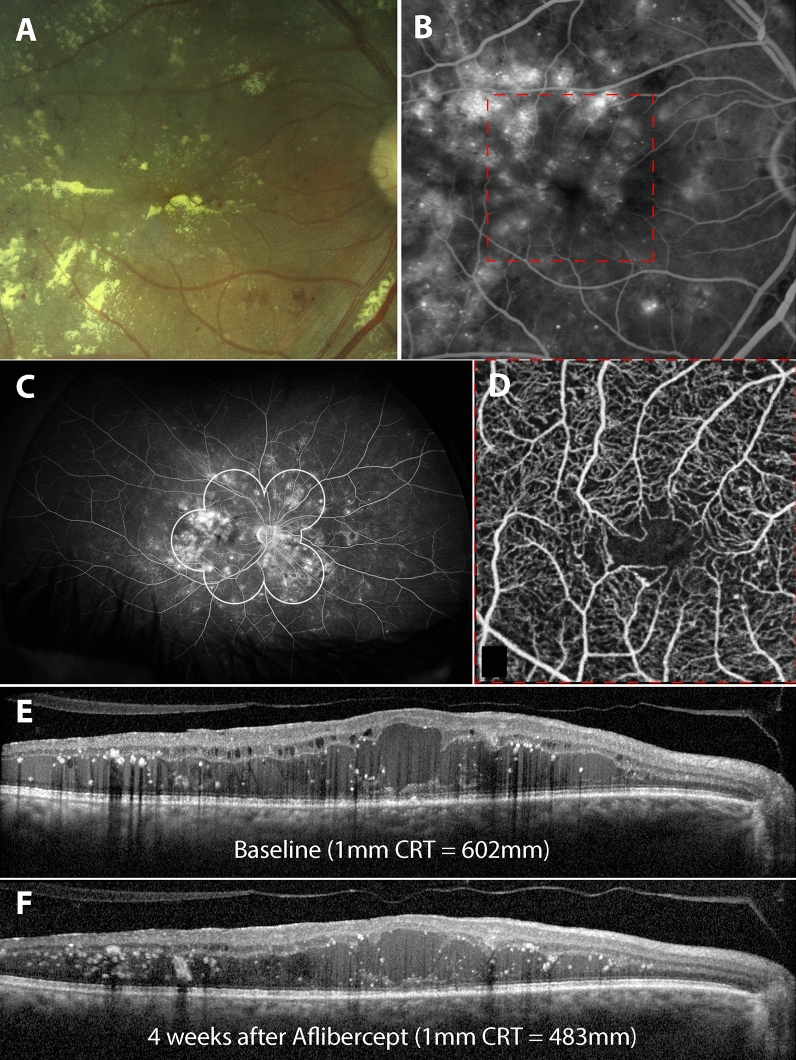
Central retinal thickness (CRT) changes in eyes without evidence of retinal non-perfusion outside the Early Treatment Diabetic Retinopathy Study (ETDRS) 7-field grid. The imaging features of a 60 year-old male with Type 2 Diabetes Mellitus is presented. (**A**), Fundus photograph (Canon CX-1). (**B**)**,** Fluorescein angiography (FA) of the macula (Heidelberg Spectralis). (**C**)**,** Ultrawide field FA (Optos California). (**D**)**,** Optical coherence tomography angiography (OCTA) of the macula (Optovue XR Avanti). (**E**), Single B-scans of the central fovea at baseline and (**F**), 4 weeks after treatment with 2 mg of aflibercept are provided (Heidelberg Spectralis). Note that there has not been as great a reduction in central retinal thickness (CRT) in this eye compared to the patient in Fig. 3. The FA demonstrates leakage at the macula but there is no evidence of non-perfusion outside the ETDRS 7-field grid. Perifoveal capillary density is mostly preserved on OCTA.

The factors associated with significant reduction in CRT included the presence of peripheral non-perfusion outside the ETDRS 7-field grid (*p* = 0.005, c = 25.0 µm) and LDL (*p* < 0.001, c = 14.7 µm). Factors that did not appear to influence CRT reduction are summarized in Table 3 and included prior VEGF/steroid intravitreal therapy, systemic co-morbidities, glycemic control and renal function. Mean baseline 1 mm CRT in the group with peripheral non-perfusion present (408.1 ± 140.7 µm) was not different to the group with peripheral non-perfusion absent (376.9  ± 110.3 µm; *p* = 0.32). The change in 1 mm CRT between final and baseline visits was greater in eyes with peripheral non-perfusion present (117.3 ± 121.4 µm) compared to eyes with peripheral non-perfusion absent (80.0 ± 72.6 µm). The change in 1 mm CRT at each visit for the two groups is summarized in Fig. 2. Significant differences in the change of 1 mm CRT at month one were seen between the two groups with peripheral non-perfusion present (102.6 ± 123.0 µm) and with peripheral non-perfusion absent (44.7 ± 46.2 µm); (*p* = 0.018), at 3 months with peripheral non-perfusion present (115.1  ± 121.9 µm) and with peripheral non-perfusion absent (63.4  ± 54.6 µm); (*p* = 0.040) and at 4 months with peripheral non-perfusion present (129.7 ± 128.2 µm) and with peripheral non-perfusion absent (61.7 ± 57.9 µm); (*p* = 0.018). In both groups, the greatest reduction in 1 mm CRT occurred in the first month. We also analyzed the changes in 3 mm and 6 mm CRT and found similar relationships consistent with the trends seen in 1 mm CRT. There was no difference in BCVA change between baseline and final visits between groups with peripheral non-perfusion present and absent (minimum *p* > 0.38; Fig. 2). Illustrative cases that demonstrate the association between retinal imaging findings and response to IAI in groups with peripheral non-perfusion present (Fig. 3) and absent (Fig. 4) on the UWF FA have been provided.

Patients above and below an LDL value of 2.6 mmol/L demonstrated a reduction in CRT post treatment. However, the reduction was greater in eyes with LDL above 2.6 mmol/L when compared to eyes below 2.6 mmol/L LDL from a normalized baseline (Fig. 2).

No measures of baseline FAZ shape or size including area, perimeter, maximum and minimum diameters, axis ratio, eccentricity, or acircularity index revealed any significance for BCVA improvement or CRT reduction.

There was no significant relationship/correlation between macular VD and peripheral non-perfusion.

## Summarized results (Table 3)

### Predictors at baseline of BCVA improvement at 6 months

- Higher macular VD ≥ 0.1 as quantified by OCTA (*p* = 0.001)
- LDL ≥ 2.6 mmol/L (*p* = 0.017)
- T1DM (*p* = 0.014)

### Predictors at baseline of CRT reduction at 6 months

- Presence of peripheral non-perfusion as graded on UWF FA (*p* = 0.005)
- LDL ≥ 2.6 mmol/L (*p* < 0.001)

### Predictors at baseline of excellent BCVA (≥ 70 ETDRS letter score) at 6 months

- Male sex (*p* = 0.004)
- LDL ≥ 2.6 mmol/L (*p* < 0.001)
- T1DM (*p* = 0.002)
- Intact ELM/EZ (*p* = 0.034)
- Higher macular VD ≥ 0.1 as quantified by OCTA (*p* = 0.002)

## Discussion

The prevalence of DM is continuing to rise and is projected to affect nearly 700 million people by 2045^23^. The rise in the global prevalence of DM will be paralleled by an increase in the vision threatening complications of DR such as DME. Although intravitreal anti-VEGF therapy has proved to be an effective treatment for DME, a considerable number of patients have sub-optimal response to this therapy. The post hoc analysis of the Diabetic Retinopathy Clinical Research Network Protocol I study revealed that nearly 40% of eyes gain < 5 ETDRS letters after 3 months of intravitreal ranibizumab therapy and approximately 50% of these maintain poorer long term visual outcomes^3^. The study by Dinah and colleagues revealed that 52% of eyes treated with IAI over 12 months for DME (mean number of injections being 6.2) achieved < 100 µm reduction in CRT^24^. Collectively, the clinical evidence implicates a subset of patients with DME that are poor candidates for anti-VEGF therapy. To date, pre-treatment identification of this subset of patients has been difficult.

Systemic factors play a major role in modulating the natural course of DR. Case controlled clinical trials have shown that duration of DM, glycemic control, hypertension and dyslipidemia are significant predictors of DR development and progression^25^. There is growing evidence to support the association between diabetic nephropathy and the development of the proliferative retinal complications^26^. Although DME can develop at any stage of DR, recent lines of evidence suggest that ocular factors play a predominant role in predicting the development of DME compared to systemic factors. Martinho et al. performed a 5-year prospective longitudinal observational cohort study to investigate the ocular and systemic risk factors for the development of DME^27^. They found that microaneurysm turnover and ganglion cell layer and inner plexiform layer thickness metrics, as measured using OCT, were better predictors for the development of macular edema than systemic markers of metabolic control. However, it remains unclear if ocular biomarkers are more informative than systemic risk markers for predicting response to anti-VEGF in the setting of DME.

Retinal imaging plays a key role in reconciling treatment strategies for DME. Sophie et al. evaluated the predictors of functional and anatomic outcomes in patients with DME treated with ranibizumab^15^. They found that OCT-derived biomarkers such as subfoveal fluid and intraretinal cystoid changes were significant predictors of visual outcomes. However, in that report, OCTA and UWF FA was not performed or correlated with outcomes. Fan and colleagues used data from the DAVE study to show that retinal vascular bed area (the total retinal vascular bed area as seen on stereographic projection on UWF FA) was correlated with the severity of DME^28^. Their study however did not take into the account key systemic variables in their predictive modelling. A major strength of our study is that we performed contemporaneous retinal imaging using state-of-the art technology including high-resolution OCT, OCTA and UWF FA at the baseline visit. With comprehensive grading of these images we included imaging biomarkers that have previously been shown to influence the natural course and treatment response in DME. We also considered the role of key systemic factors such as glycemic control, dyslipidemia and renal function in treatment outcomes; therefore, our study was designed to account for potential interactions between imaging and systemic variables.

The relationships between peripheral and central retinal changes in DR are complex but inherently linked. The occurrence of peripheral retinal non-perfusion is known to increase the risk of central macular complications such as DME^28^. Hypoxia and retinal non-perfusion are potent stimulants for VEGF production. However, the magnitude and effects of VEGF upregulation following non-perfusion in the central retina and the peripheral retina are unlikely to be equivalent. This might be due to the significant topographic variations in the density, distribution and subtype of VEGF-producing cells within the retina including retinal ganglion cells, retinal endothelium, glia and retinal pigment epithelial cells. In our study, the occurrence of peripheral non-perfusion was a significant predictor of anatomic response to IAI whilst macular non-perfusion, as quantified using OCTA VD measurements, was not found to be a significant predictor. This finding may suggest that VEGF upregulation is significantly greater in the setting of peripheral non-perfusion than macular non-perfusion in DR. The greatest reduction in 1 mm CRT (25% CRT reduction in first month) occurred following the first treatment in eyes with peripheral non-perfusion compared to those eyes where peripheral perfusion appeared to be preserved (12% CRT reduction in first month). The change in CRT between baseline and month 6 was also greater in the non-perfusion group. It should be noted that eyes with preserved peripheral retinal perfusion also manifested a significant reduction in CRT following the first treatment, but it was not as great as the non-perfusion group. In such eyes, it is likely that additional pathogenic mechanisms, such as inflammation, are playing a role in DME as opposed to predominantly an ischemic driven phenomenon.

The introduction of OCTA has facilitated clearer delineation of the association between macular vascular biomarkers and visual outcomes in retinal vascular diseases. In our previous report, we showed that the area of the FAZ is correlated with BCVA in DR and retinal vein occlusion^29^. Endo et al.^30^ showed that FAZ circularity was significantly correlated with BCVA in 183 eyes of patients with DM. A major advantage of OCTA is that it permits clear visualization of retinal capillaries that are the major sites of nutrient and waste exchange in the retina. Advances in image analysis software and algorithms has allowed quantification of OCTA capillary density measurements as a means of detecting macular disease and monitoring disease progression. Using our previously validated technique we have shown in the present study that macular VD measurements are the strongest predictor of BCVA following treatment with IAI.^31^ In contrast to previous reports, FAZ area was not found to be a significant predictor in this study after accounting for the influence and interactions of VD. Previous investigators have highlighted the relationship between capillary density and BCVA in DR. Dupas and colleagues imaged 22 eyes of patients with Type 1 DM using OCTA and showed that a reduction in BCVA was associated with a reduction in vascular density in the deep capillary plexus^32^. Samara et al.^33^ showed a significant negative correlation between BCVA and vascular density in the superficial and deep networks in patients with DR. In both of those reports patients with DME were excluded. The mechanism by which VD can influence BCVA is unclear. One explanation might be that VD is a surrogate marker of the function of macular amacrine and horizontal cells, both of which play a key role in regulating photoreceptor homeostasis. Using transgenic mice, Usui et al.^34^ showed that amacrine cells play a vital role in promoting photoreceptor function through vascular maintenance of the intermediate plexus. They also showed that loss of the intermediate plexus accelerated retinal degeneration. This postulation requires further investigation in the setting of DME.

Our study also provides evidence of a threshold-based relationship between VD and BCVA in DME. We show that patients with macular VD measurements less than 0.1 did not have a significant improvement in BCVA over time despite a significant reduction in CRT following IAI therapy. Such a threshold in VD may denote a point of critical photoreceptor depletion beyond which BCVA cannot be improved with anti-VEGF therapy. Accordingly, in addition to VD, we show that the greatest predictor of excellent visual outcome (BCVA 20/40 or better) at the final visit was the presence of an intact EZ/ELM band at the baseline visit. As the EZ represents a structurally intact photoreceptor band it is not surprising that its integrity is correlated with BCVA as per previous studies^29,35^.

Dyslipidaemia modulates the natural course of DR. Treatment of dyslipidemia with fenofibrates can alter the natural course of DR and the development of complications. In a recent report, Meer et al. showed that use of fenofibrates in patients with non-proliferative DR was associated with a significantly decreased risk of developing proliferative DR (hazard ratio, 0.76). However, in the same study, the use of fenofibrates did not reduce the risk of developing DME (hazard ratio, 0.96)^36^. Oxidized LDL can promote atherosclerosis through inflammatory and immune-complex mediated damage to the arterial intima^37^. Diabetes can amplify LDL oxidation and damage retinal vessels in a manner that is similar to atherogenesis. Using human donor eyes from patients with DM, Wu et al. showed that oxidized LDL played a major role in mediating apoptosis of retinal cells including pericytes^38^. From a clinical standpoint, there is evidence to suggest that dyslipidemia can modulate the onset and complications of DR. In the meta-analysis performed by Zhou and colleagues, LDL was found to be the only sub-type of cholesterol that was significantly increased in patients with DR^39^. In a separate systematic review, Das et al. found that total serum cholesterol, LDL and serum triglycerides were significantly higher in patients with DME compared to those without DME^40^. The present study provides evidence that dyslipidemia predicts treatment response to IAI in DME. We show that patients with elevated LDL (≥ 2.6 mmol/L) had significantly greater visual gains as well as significantly greater reduction in CRT following treatment with IAI. High density lipoprotein levels were not found to be a significant predictor of these outcomes. We emphasize that baseline VA and CRT was not significantly different between patients with LDL < 2.6 mmol/L and those with LDL ≥ 2.6 mmol/L. There is evidence to suggest that VEGF protects against oxidized LDL-mediated endothelial damage therefore it is difficult to explain how antagonizing the action of VEGF through IAI can reduce CRT or improve visual outcomes in DME^41^. However the study by Ladowska et al.^42^ on neovascular age-related macular degeneration found a similar association to our report. They found that patients with LDL hypercholesterolemia had significantly greater reductions in CRT following intravitreal ranibizumab and IAI for neovascular age-related macular degeneration. The manner by which LDL modulates the anatomic and visual outcomes following anti-VEGF therapy therefore requires further investigation.

Limitations of this report include the sample size of which 76 eyes were included, it is possible this report may function as a pilot for larger investigations. To achieve this sample size, we included patients who were previously treated, with laser or anti-VEGF therapy, we accounted for this by having a minimum of 6-month period between their last treatment and baseline imaging. This wash out period defined in this study is greater than what is incorporated in many clinical trials in the field of DME^43^. Another perceived limitation of the study is the inclusion of patients with good baseline VA. Although the results of the Protocol V study did not demonstrate a significant difference in vision loss between prompt anti-VEGF and observation for the treatment of DME it was not designed to determine if early treatment reduced the risk of vision loss in such eyes^44^. The ETDRS study demonstrated a significant benefit in reducing risk of vision loss following early treatment of DME and that was the rationale for including patients with good visual acuity in the study^45^. We only included patients that demonstrated progression of DME on OCT and therefore were deemed to be at risk of vision loss without treatment. Other postulated arguments for early treatment of DME is to reduce the risk of neurodegeneration that may be secondary to persistent intraretinal fluid^46^.

In this report we performed a comprehensive analysis of imaging, demographic and systemic factors to demonstrate that retinal vascular biomarkers are useful and significant predictors of functional and anatomic outcomes following IAI therapy for DME. As it is possible to quantify macular vascular density and retinal non-perfusion with increasing precision the findings of this work have practical applications for the real-world management of DME. The biomarkers discovered here may be used to better-select responders to IAI therapy, choose patients who may be better suited to intravitreal corticosteroids as a first-line therapy or support an earlier switch to alternative anti-VEGF therapies or steroid treatments if IAI therapy has already commenced and to more accurately counsel patients regarding long term visual outcomes. Further work will be required to determine if the findings of this prospective study have relevance for predicting treatment response for other anti-VEGF agents including ranibizumab, bevacizumab and faricimab. An important future direction of this research would be replicate these findings in a larger cohort and to further refine the quantitative relationships between the magnitude of retinal non-perfusion and CRT reduction following therapy. Fan et al. and others have described novel ways of quantifying ischemic index and retinal perfusion area using UWF FA^47^. We did not perform such techniques in this report as we lacked access to proprietary software that allowed conversion of UWF FA images into stereographic projections. Applications of such techniques could potentially result in an even more nuanced approach for predicting the magnitude of treatment response to IAI. Such quantitative techniques for measuring peripheral non-perfusion and OCTA-based macula VD measurements may be readily applied to algorithms that can assist clinicians stratify patients into categories based on expected treatment response. This individualized selection of patients who would benefit from IAI could ultimately serve to reduce the global burden due to DME and expedite favorable outcomes.

## Methods

This study followed the Tenets of the Declaration of Helsinki and was approved by the Human Research Ethics Committee at The University of Western Australia. Data was stored and managed in compliance with guidelines from the Health Insurance Portability and Accountability Act. The trial was registered with the Australian New Zealand Clinical Trials Registry (ACTRN12621001371886) dated 11/10/2021.

### Subjects

Patients were prospectively recruited from the Eye Clinics of the Lions Eye Institute and Sir Charles Gairdner Hospital in Perth, Australia between March 2019 and February 2021. Informed consent was obtained. Eligible patients were at least 18 years of age with Type 1 or 2 DM with DME assessed to be the cause of visual symptoms. Patients with good BCVA were included in this study if there was documented progression of DME on OCT as determined by a progressive increase in the 1 mm central retinal thickness over 6 months and the patient was judged to be at risk of vision loss if left untreated^48^. Patients with treatment-naïve or previously treated DME and retinal thickness measured on OCT ≥ 250 µm in the central subfield were enrolled. Exclusion criteria included (1) any concomitant ocular disease that causes macular edema such as age-related macular degeneration or retinal vein occlusion, (2) any other ocular condition that was assessed to compromise BCVA, (3) previous treatment with intraocular corticosteroids, intravitreal anti-VEGF agents or laser photocoagulation therapy (macular or pan-retinal photocoagulation; PRP) within 6 months of the baseline visit^43^, (4) intraocular Pressure ≥ 21 mmHg at baseline visit and (5) poor quality retinal imaging that precluded qualitative or quantitative analysis. A patient could have two study eyes included if both were eligible at baseline visit.

Patients were not subject to any other ocular intervention such as retina laser during the period of enrolment in the study. Additionally, all patients in this study had well-controlled and stable blood pressure during the duration of enrolment as ascertained by the records of their family physician. There were no medication changes for the treatment of DM, hypertension or dyslipidemia in any of the patients during the period of enrolment.

### Data collection at baseline visit

At baseline all patients underwent subjective refraction and BCVA measurement as per the ETDRS protocol^49^. Demographic and clinical information, including treatment history and duration of disease, was obtained for each subject. Within one week of the baseline visit, patients had blood collected for assessment of glycated hemoglobin (HbA1c), renal function including creatinine and glomerular filtration rate and fasting lipid profile. Medical records were examined for any history of hypertension, cardiovascular disease (stroke or myocardial infarction), smoking history, use of any lipid lowering therapy (statins, fibrates, ezetimibe) and ocular history (past intravitreal VEGF or steroids, phakic status, past vitrectomy). Table 1 summarizes all non-imaging baseline features that were recorded. Retinal imaging at baseline visit followed a standardized protocol and included the following:

1. *Color fundus photography* Retinal photographs of the posterior pole and macula were acquired using the Canon CX-1 digital retinal camera (Retinal Imaging Control Software for CX-1, 4.6.0.5, Canon Medical Systems, Otawara, Japan). UWF retinal photographs were captured using the Optos California [Optos 200Tx (Optos 200Tx; Optos, Dunfermline, Scotland, United Kingdom].
2. *Spectral-domain OCT* OCT images were acquired using the Heidelberg Spectralis OCT2 device (Heidelberg Engineering, Heidelberg, Germany). A raster scan protocol centered at the fovea (range 30° × 25°) comprising 61 B-scans (122 µm interscan distance) with automatic real-time tracking (ART) mode enabled (9 B-scan images averaged) was acquired. OCT images at each visit were acquired such that the volume scan was registered to the previous visit. This allowed direct point-to-point comparisons of OCT features between each visit to follow up progression and/or treatment response.
3. *Fluorescein angiography* Multiple frames of the initial 5 min of the angiogram sequence were captured using the Heidelberg Spectralis OCT2 Module (Heidelberg Eye Explorer, 1.10.4.0, Heidelberg Engineering, Heidelberg, Germany). The scan angle was 30 degrees with ART mode enabled (22 images averaged). Multiple frames of UWF FA images including the retinal peripheries were then captured for the period that was 5—10 min after fluorescein injection using the Optos California (Optos 200Tx; Optos, Dunfermline, Scotland, United Kingdom) which can capture up to 200° of retina.
4. *Optical coherence tomography angiography* OCTA images of the macula were acquired using the Optovue XR Avanti (XR Avanti RTVue XR. 2018.1.0.43, Optovue, Inc., Freemont, CA) and / or Heidelberg Spectralis OCT2 Module (Heidelberg Eye Explorer, 1.10.4.0, Heidelberg Engineering, Heidelberg, Germany).

The Optovue XR Avanti obtains split spectrum amplitude decorrelation angiography images. This instrument has an A-scan rate of 70,000 scans per second, using a light source centered on 840 nm and a bandwidth of 45 nm. The scan area was 3 × 3 mm centered on the fovea. Each OCTA volume is acquired in approximately 3 s and two orthogonal OCTA volumes were acquired to perform motion correction and minimize motion artifacts arising from micro-saccades and fixation changes. Angiography information displayed is the average of the decorrelation values when viewed perpendicularly through the thickness being evaluated. If the image processed with motion correction software demonstrated artifact involving the foveal avascular zone (FAZ) in the form of doubling of vascular structures or sideways shearing, then the case was excluded from analysis. Segmentation of retinal vascular layers was not performed for this study as the presence of macular edema significantly affects the software’s accuracy of automatic layer segmentation. Only OCTA images with signal strength above 50 were included in this study.

The Heidelberg Spectralis OCT2 device uses a full-spectrum probabilistic approach. This instrument has an A-scan rate of 85,000 scans per second, using a light source centered on 870 nm and a bandwidth of 50 nm. A total of 512 B scans (6 µm interscan distance) with ART mode enabled (4 images averaged) was used to obtain 3.1 × 3.1 mm OCTA volume scans (10º × 10º) centered at the fovea. Images were acquired with Projection Artifact Removal enabled.

With each OCTA device we aimed to capture 8—10 consecutive OCTA scans within a period of 5 min from the study eye. When multiple high-quality OCTA scans were captured, we performed averaging of these scans as previously described to improve signal noise ratio and generate a single projection vascular slab representing all the retinal vascular layers^50^. Image projections were prepared using Fiji ImageJ2 (ver. 2.30/1.53f.: https://imagej.net, originally developed by Wayne Rasband, National Institutes of Health, Bethesda, MD).

1. *Axial length measurement and keratometry* Axial length and keratometry measurements were acquired using the IOL Master 700 (Carl Zeiss Meditec AG, Jena, Germany). These measurements together with refraction were used to correct image magnification errors due to axial length variations on OCTA images as described below.

### Treatment and study procedure

After eligibility was determined, study eyes were treated with IAI (2.0 mg in 0.05 mL, Bayer, Australia). Each patient received 6 total injections that included 5 treatments separated by an interval of 4 weeks with the 6th treatment given between 4 and 8 weeks after the 5th treatment. At each visit, subjects underwent BCVA measurement by the ETDRS protocol. Color photography, OCT and OCTA imaging as described above was performed at each monthly visit.

### Image analysis

Retinal imaging from the baseline visit was assessed independently by two experienced retina specialists (IJC and VR) in a masked fashion. A third retina specialist (CB) reconciled any disagreement in grading. Multimodal imaging was used to assess the following features:

1. *Presence and number of microaneurysms in the macula* A combination of color photography, OCT and FA images were used to define microaneurysms (Supplementary Fig. 1). The utilization of multiple imaging modalities allowed differentiation of microaneurysms from retinal hemorrhages. The macula (diameter 5.5 mm) was assessed for the occurrence of microaneurysms. In this area, microaneurysms were counted and graded as being either: absent, less than 10 microaneurysms in number or greater than 10 microaneurysms in number. The rationale for assessing microaneurysms is the evidence for the association between the microaneurysm lifecycle and the risk of progression of DME^51–53^.
2. *Presence or absence of exudate in the macula* Color photography and OCT were used to define exudation and differentiate it from hyperreflective foci (HRF) (Supplementary Fig. 2). The macula (diameter 5.5 mm) was assessed and exudation was graded as being present or absent. The rationale for assessing the presence of macular exudation is the association between the macular exudation and visual outcomes in DME^54^.
3. *Pattern of fluorescein leakage in the macula* Angiogram images from the Heidelberg Spectralis OCT2 were assessed. Angiographic leakage patterns in the macula were characterized using the definitions provided by the ETDRS study into focal, intermediate or diffuse leaks (Supplementary Fig. 3)^55^. Each eye was then graded according to the following definitions. Eyes with focal leak were defined as those with fluorescein leakage predominantly (> 67%) from microaneurysms (Supplementary Fig. 3A–C). Eyes with diffuse leak were defined as those with leakage mainly from dilated capillaries (< 33% from microaneurysms; Supplementary Fig. 3D–F). Eyes with intermediate leak was defined as those where leakage occurred equally from microaneurysms and dilated capillaries. The rationale for evaluating macular angiographic leakage patterns is previous work suggesting that anti-VEGF therapy has increased efficacy in the management of DME due to diffuse leaks^56^.
4. *Presence/absence of non-perfusion outside the ETDRS 7-field grid***:** An overlay of the ETDRS 7-field grid on a UWF FA scan was used to judge the presence or absence of non-perfusion outside the grid (Supplementary Fig. 4). Images were assessed in a binary fashion (present or absent) for the occurrence of any degree of peripheral non-perfusion. The rationale for evaluating UWF FA is the evidence for the association between retinal peripheral non-perfusion outside the ETDRS 7-field grid and the severity of DME^47^.
5. *Structural OCT volumes of the macula* were assessed for features that have previously been shown to predict intravitreal treatment response and visual outcomes in DME^15,29,57,58^. Each OCT volume was assessed for the presence of the following features (Supplementary Fig. 5):
6. Presence/absence of subretinal fluid.
7. Presence/absence of cysts in the inner nuclear layer (INL).
8. Presence/absence of cysts in the outer nuclear layer (ONL).
9. Presence of disorganization of retinal inner layers (DRiL). DRiL was defined as areas for which any boundaries between the ganglion cell-inner plexiform layer complex, INL, and ONL could not be identified.
10. Disruption of the external limiting membrane (ELM) and ellipsoid zone (EZ). The central 5.5 mm of the macula was evaluated. Disruption of the EZ and ELM was categorically graded as being present or absent. A ‘present’ grading was denoted if any degree of disruption to the EZ or ELM was evident.
11. Presence/absence of intraretinal HRF (Supplementary Fig. 2).
12. OCT derived measurements of retinal thickness. The 1 mm, 3 mm and 6 mm CRT was recorded from the retinal thickness ETDRS grid generated by the Spectralis Software (Heidelberg Engineering, Heidelberg, Germany; Supplementary Fig. 5B).
13. *OCTA images* were assessed for features that have previously been shown to predict treatment response to anti-VEGF therapy and visual outcomes in DME^29,32,59^. OCTA slabs including all vascular layers were used for analysis. Segmentation of the superficial vascular complex and deep capillary plexus was not performed due to unreliable segmentation of capillary plexus boundaries in the setting of DME^60^. Image-averaged projections were used for analysis in 55 of the 76 eyes. In those eyes where it was not possible to achieve image averaging using multiple OCTA frames the single best OCTA frame was used for analysis. Images were graded for the following features (Supplementary Fig. 6):
14. Perifoveal capillary loss—This was categorically graded as being present or absent.
15. Integrity of the terminal foveal capillary ring—This was categorically graded as being intact or disrupted.
16. Macular vessel density—Axial length, keratometry values and refraction were used to scale OCTA images. Our previously reported automated technique was then used to determine macular capillary density (Supplementary Fig. 7). The multi-step OCTA image processing methods used for this study were implemented using MATLAB as follows. First, the *en face* OCTA images were segmented using a deep neural network framework^61^. The resulting vessel segmentations were binarized at an intensity threshold of 0.5, where pixel intensity values of the deep neural network segmented image range from 0 to 1. Quantification metrics including macular vessel density (VD) and FAZ characteristics were extracted from these binarized images. To quantify VD, firstly perfusion density was calculated as the ratio of total pixels segmented as vasculature divided by the total number of pixels per image. VD was then calculated as the skeletonized version of perfusion density. VD was chosen over perfusion density as it is independent of vessel width and thus a more accurate measure of macular capillary density. Perfusion density is dependent on segmented vessel width, a characteristic that varies with hardware, segmentation method and segmentation binarization threshold chosen, making it a less reliable measure of capillary density (Supplementary Fig. 7). Additionally, as minimal nutrient exchange takes place at the site of large retinal vessels, the use of VD provides a more accurate quantitative measure of the macular vasculature that is involved in metabolic exchange. FAZ characteristics and dimensions were quantified by their area, perimeter, maximum and minimum diameters, axis ratio, eccentricity, and acircularity index. All OCTA measurements were further refined by correcting for image magnification errors using each eye’s axial length, refraction and the Littman and modified Bennett formula^62^.

## Statistical analysis

Univariate analyses with unpaired t-tests and Wilcoxon signed-rank tests were utilized to identify significant differences in the observed continuous variables. Linear mixed-effects models employing a backwards elimination statistical process were then used to identify significant associations between explanatory and multiple response variables. The explanatory variables were as follows: hard exudates, microaneurysms, pattern of fluorescein leakage in macula, peripheral non-perfusion seen outside the ETDRS 7-field grid, presence of subretinal fluid, occurrence of cystoid changes in the INL and ONL, presence of DRiL, integrity of EZ, presence of intraretinal HRF, perifoveal capillary loss, integrity of the terminal foveal capillary ring, macular VD, age, sex, insulin use, Type 1 or 2 DM, HbA1c, estimated glomerular filtration rate, creatinine, smoking status, low density lipoprotein (LDL), high density lipoprotein, myocardial infarction, stroke, PRP, previous anti-VEGF therapy, previous intravitreal steroids, phakic/pseudophakic and previous vitrectomy. The key response variables were CRT and BCVA change calculated as the delta between baseline CRT or ETDRS letter score and each monthly time point. Quantitative OCTA characteristics of FAZ (area, perimeter, maximum and minimum diameters, axis ratio, eccentricity, and acircularity index) and macular VD were included in the same analysis. Data was presented in 95% mean confidence interval error bars for CRT and BCVA trends over time.

## Supplementary Information

Supplementary Information.

## Data Availability

The datasets generated during and/or analyzed during the current study are available from the corresponding author on reasonable request.

## Abbreviations

ART: Automatic real-time tracking
BCVA: Best corrected visual acuity
CRT: Central retinal thickness
DM: Diabetes mellitus
DME: Diabetic macular edema
DR: Diabetic retinopathy
DRiL: Disorganization of the retinal inner layers
ELM: External limiting membrane
ETDRS: Early treatment diabetic retinopathy study
EZ: Ellipsoid zone
FA: Fluorescein angiography
FAZ: Foveal avascular zone
HbA1c: Glycated hemoglobin
HRF: Hyper-reflective foci
IAI: Intravitreal aflibercept injection
INL: Inner nuclear layer
LDL: Low density lipoprotein
OCT: Optical coherence tomography
OCTA: Optical coherence tomography angiography
ONL: Outer nuclear layer
PRP: Pan-retinal photocoagulation
UWF: Ultrawide field
VD: Vessel density
VEGF: Vascular endothelial growth factor
