# Supplementary material for: Angiographic biomarkers are significant predictors of treatment response to intravitreal aflibercept in diabetic macular edema
Source: Sci Rep. 2023 May 19;13:8128. doi: 10.1038/s41598-023-35286-2 (PMC10199070; doi:10.1038/s41598-023-35286-2)
Supplement: Supplementary file 1 — Supplementary Information. [file 41598_2023_35286_MOESM1_ESM.pdf]

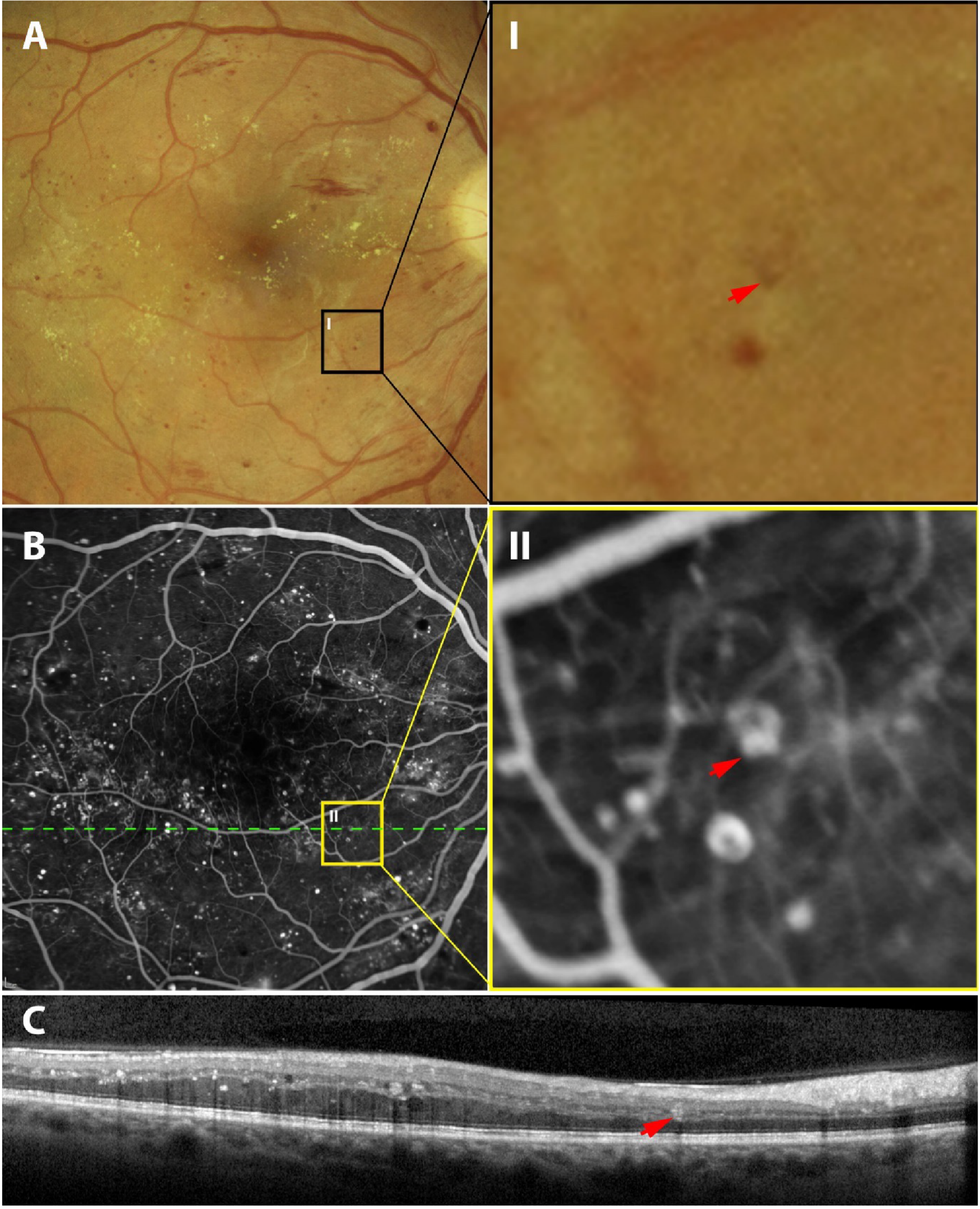

Supplementary Figure 1. Measuring retinal microaneurysms in diabetic retinopathy. Retinal microaneurysms were defined using a combination of **A**, fundus photography (Canon CX-1) **B**, fluorescein angiography (Heidelberg Spectralis) and **C**, optical coherence tomography (OCT) (Heidelberg Spectralis). The typical features of a single microaneurysm (red arrow) as seen using these 3 imaging modalities is presented. Microaneurysms were seen as red spots on fundus photography (Inset I), were hyperfluorescent on fluorescein angiography (Inset II) and demonstrated hyperreflective walls on OCT, **C**. The use of multimodal imaging allowed us to differentiate microaneurysms from retinal hemorrhages. The line of OCT imaging is demonstrated by the fenestrated green line on panel **B**.

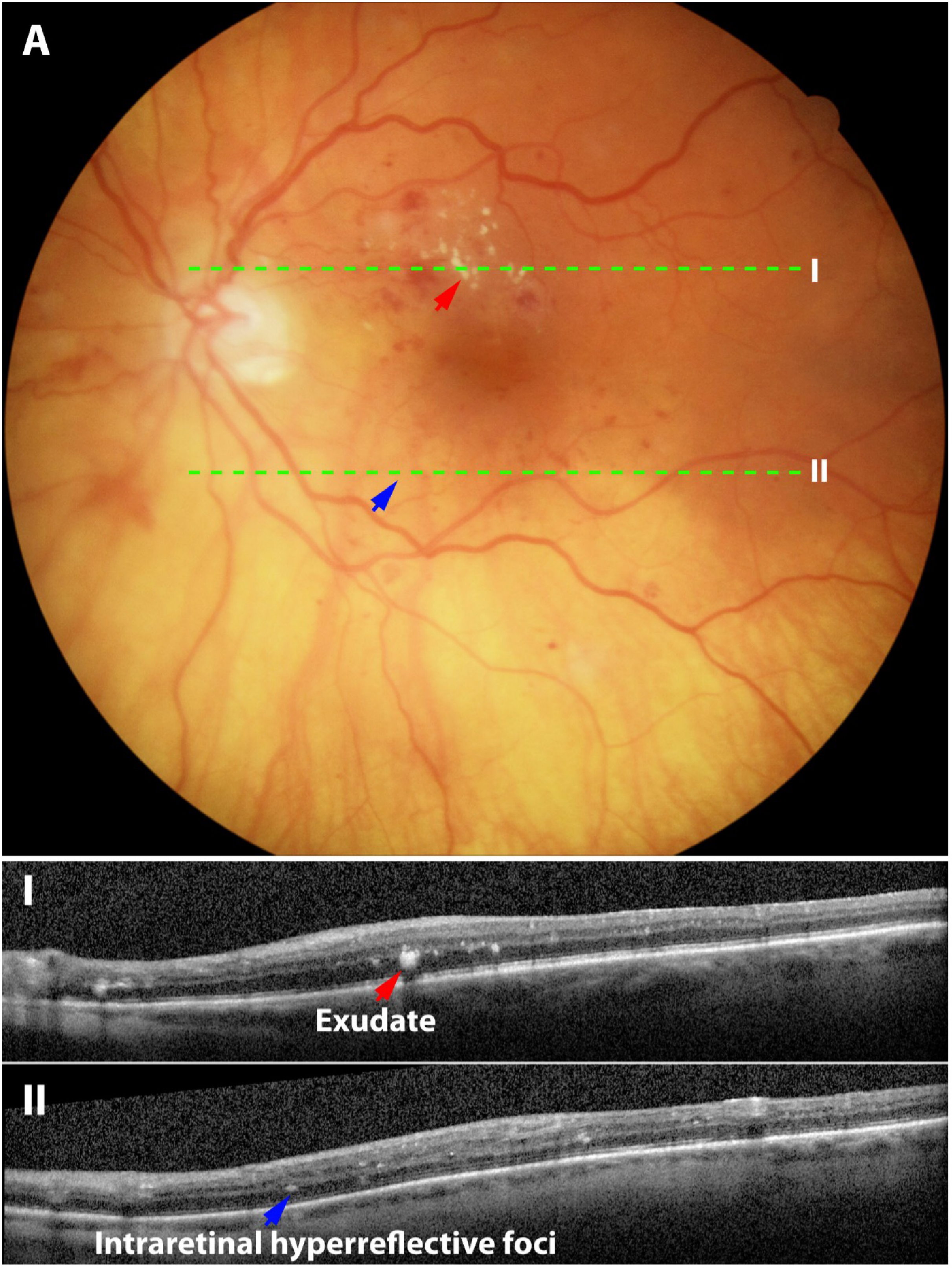

Supplementary Figure 2. Defining exudation in diabetic retinopathy. A combination of **A**, fundus photography (Canon CX-1) and **I, II**, optical coherence tomography (OCT; Heidelberg Spectralis) were used to define exudation. Exudates appeared as yellow lesions (red arrow) on fundus photography with intraretinal hyperreflective signal on OCT (**I**). This was different to intraretinal hyperreflective foci that were not seen on fundus photography but appeared as intraretinal hyperreflective spots on OCT (**II**). The point of intraretinal hyperreflective spots as seen on OCT is indicated with a blue arrow on the fundus photograph. The line of OCT imaging is demonstrated by the fenestrated green lines on panel **A**.

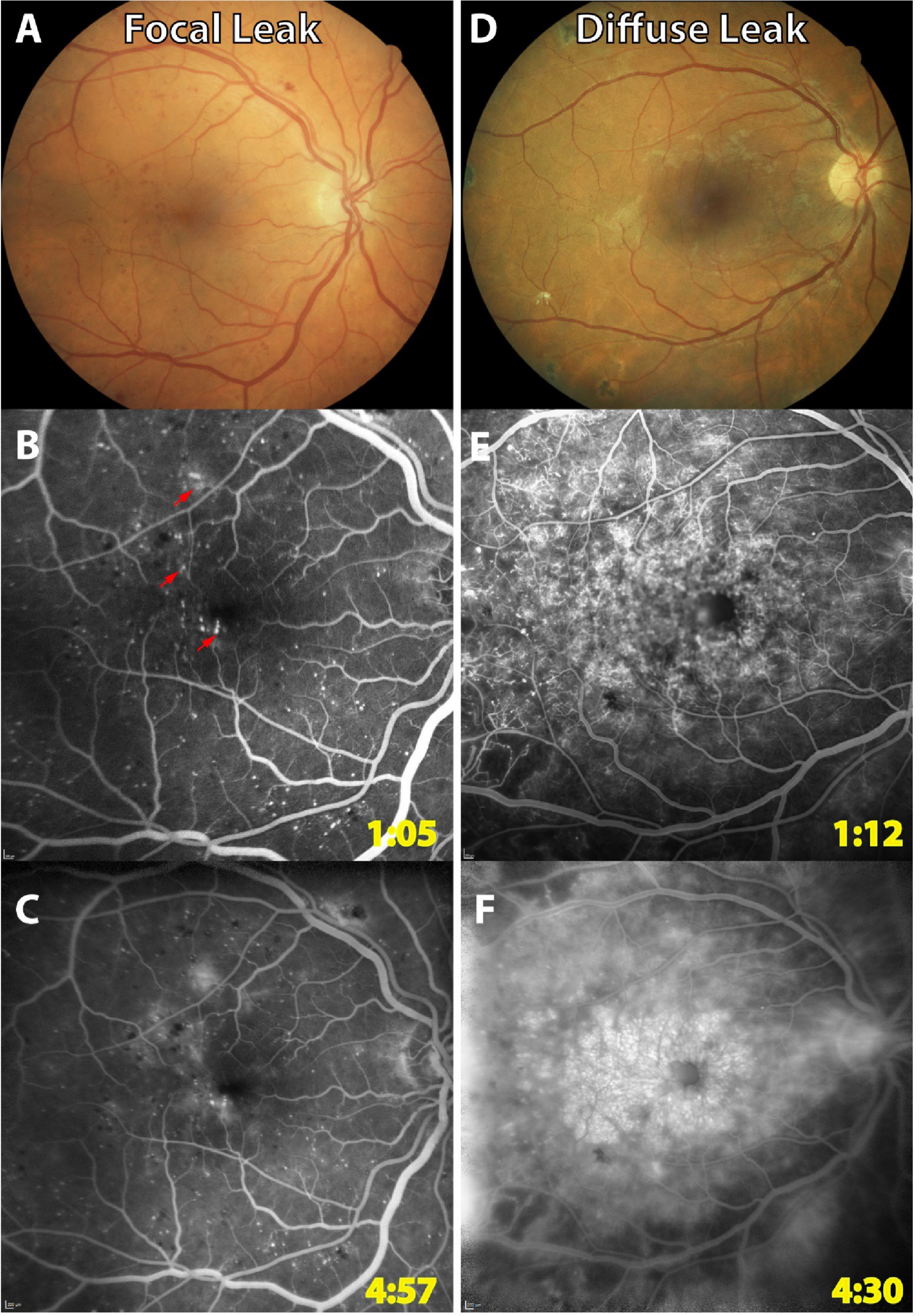

Supplementary Figure 3. Defining fluorescein angiographic leakage patterns in the macula. Contemporaneously acquired fundus photography (Canon CX-1) and fluorescein angiography (FA; Heidelberg Spectralis) images are provided. **A-C,** demonstrate the characteristics of a focal leak. Focal leaks were defined as those with leakage mainly (>67%) from microaneurysms (red arrows). Note that the areas of angiographic leakage in the late frames are largely confined to areas where microaneurysms are present. **D-F**, demonstrate the characteristics of a diffuse leak. Diffuse leaks were defined as those with leakage mainly from dilated capillaries (<33% from microaneurysms). Intermediate leaks were defined as those where leakage occurred equally from microaneurysms and dilated capillaries. Time stamps for the angiograms are provided in the lower right-hand corner of respective panels.

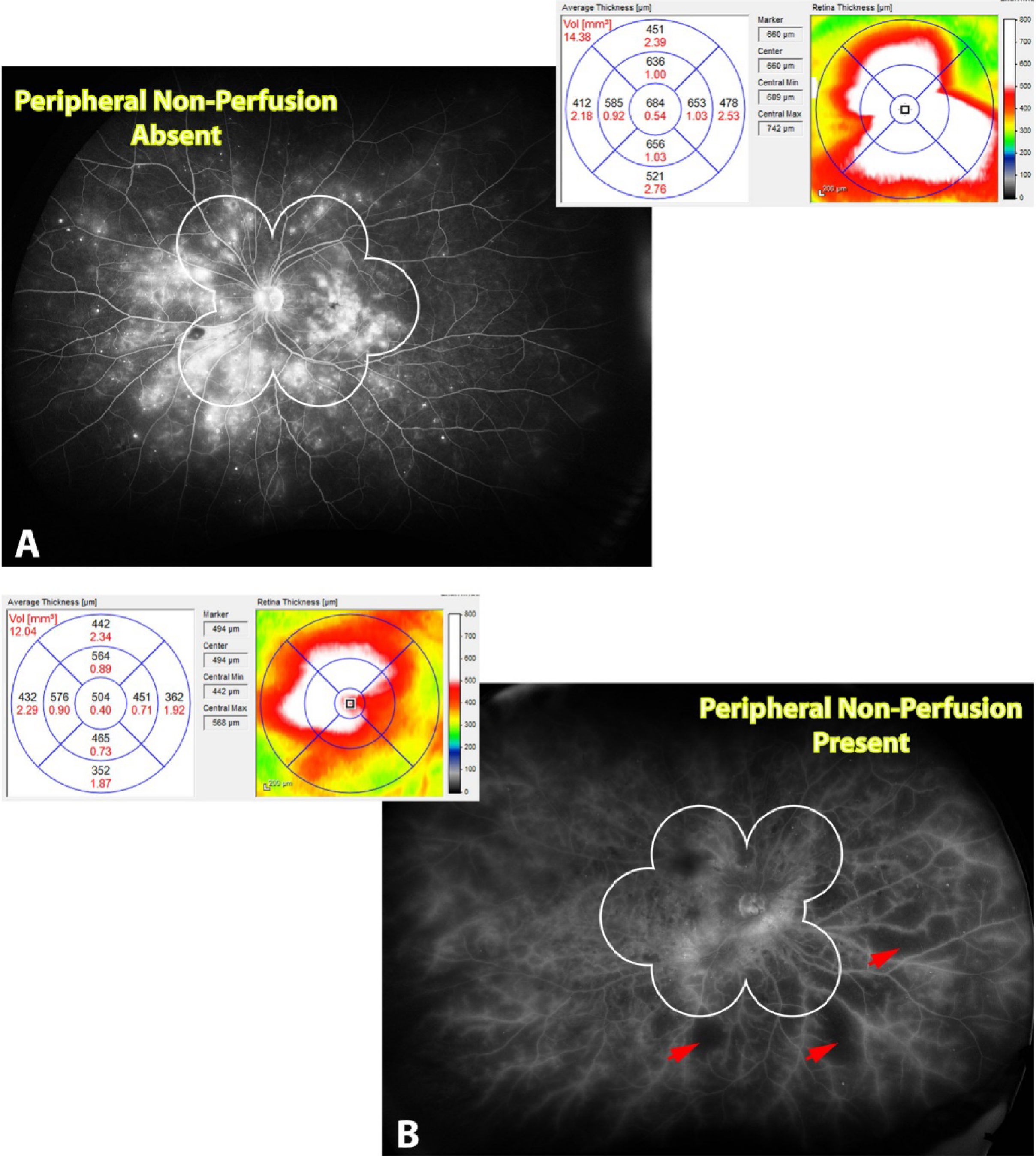

Supplementary Figure 4. Defining retinal non-perfusion outside the Early Treatment Diabetic Retinopathy Study (ETDRS) 7-field grid using ultrawide fluorescein angiography (Optos California). Examples where non-perfusion was **A**, absent and **B**, present are provided. Areas of non-perfusion (red arrows) appeared hypofluorescent or demonstrated a grayish background fluorescence that was substantially reduced compared to surrounding perfused regions. Eyes that demonstrated evidence of peripheral capillary non-perfusion also frequently demonstrated perivascular staining of peripheral retinal vessels and capillary leakage from the deep retinal circulation as shown in panel B. Note that despite significantly different ultrawide field angiographic appearances, the central retinal thickness of the two eyes are comparable as shown by the provided macular thickness maps (Heidelberg Spectralis).

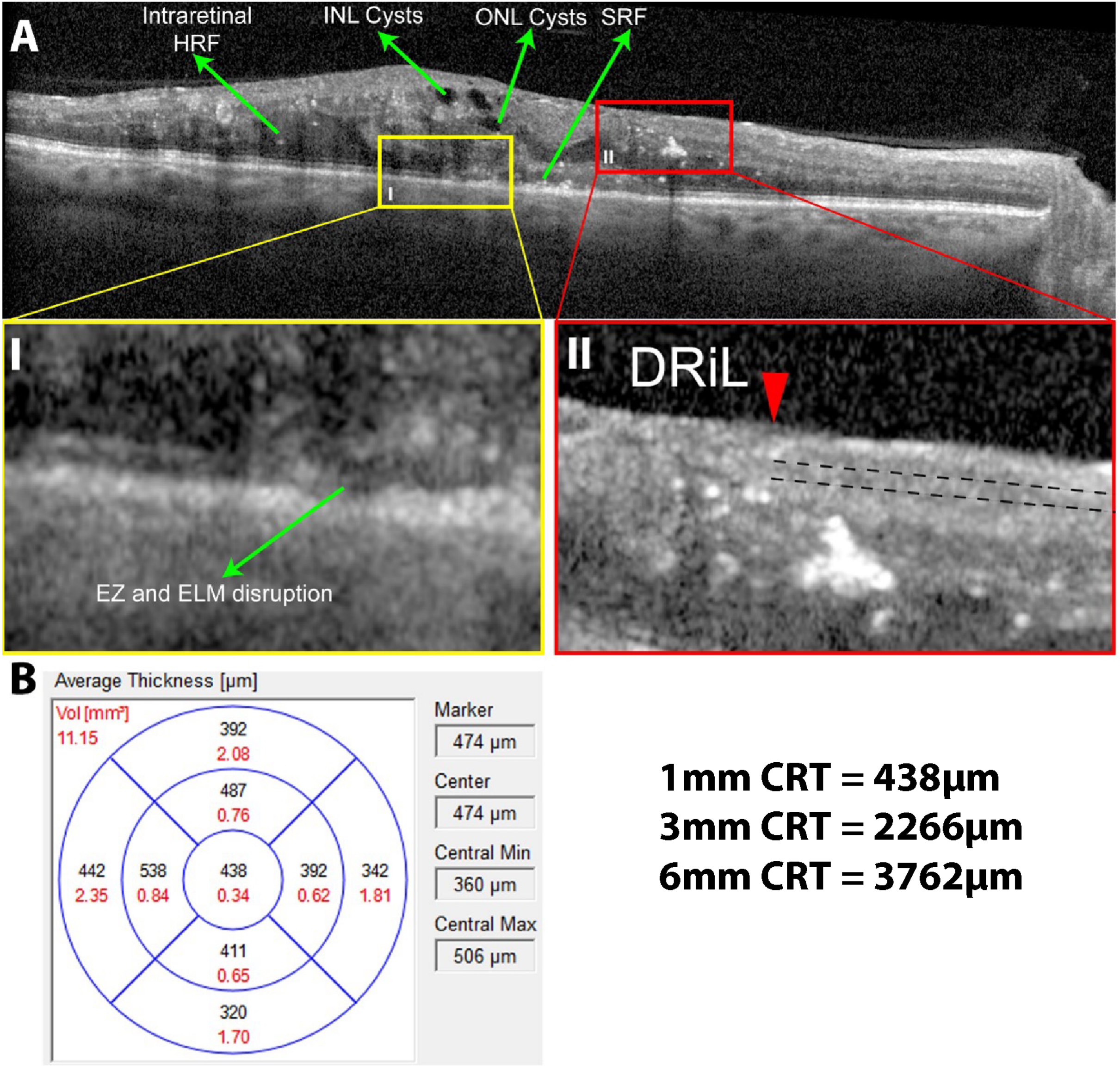

Supplementary Figure 5. Analysis of optical coherence tomography (OCT; Heidelberg Spectralis) features of diabetic macular edema. The OCT volumes were assessed for the presence of intra retinal hyperreflective foci (HRF), inner nuclear layer (INL) cysts, outer nuclear layer (ONL) cysts and subretinal fluid (SRF). Inset **I,** demonstrates the presence of ellipsoid zone (EZ) and external limiting membrane (ELM) disruption. OCT volumes were also assessed for the presence of disorganization of retinal inner layers (DRiL) as shown in Inset **II**. DRiL was defined as the horizontal extent for which any boundaries between the ganglion cell-inner plexiform layer complex, INL, and ONL could not be identified. Note that in this case the normal laminations of inner retinal layers (black dashed lines) are disturbed as the fovea is approached (red arrow) denoting the presence of DRiL. Quantitative macular thickness maps generated using **B**, Heidelberg OCT software were also used to calculate 1 mm central retinal thickness (CRT), 3mm CRT and 6mm CRT.

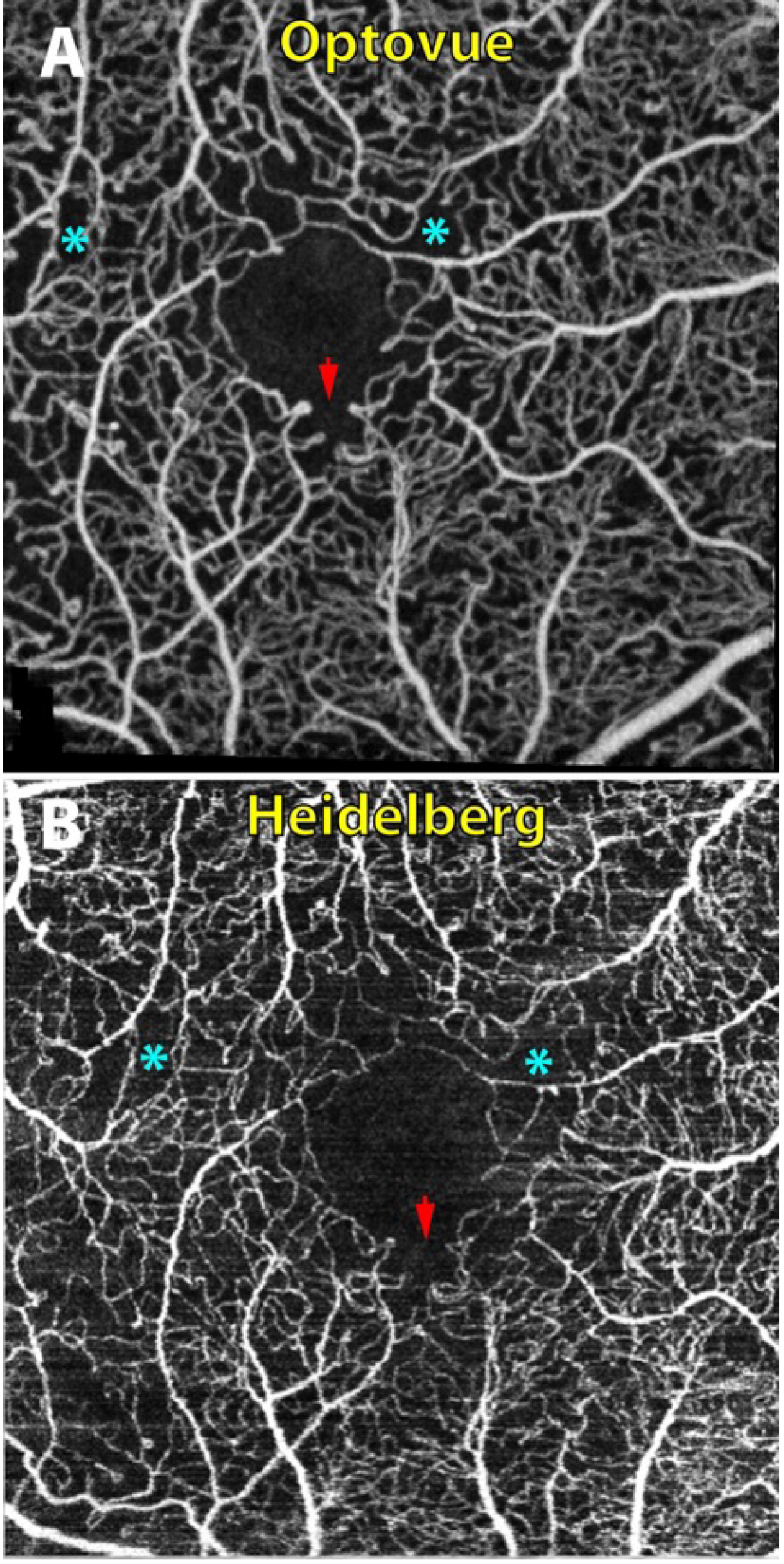

Supplementary Figure 6. Analysis of optical coherence tomography angiography (OCTA) features of the central macula. Images of the same eye from two OCTA devices including the **A**, Optovue XR Avanti and **B**, Heidelberg Spectralis are provided. Images were graded for loss of the integrity of the terminal foveal capillary ring (red arrow) and perifoveal capillary loss (asterisk). When possible, multiple OCTA images were acquired from the same eye such that image averaging could be performed as shown in **A**. This resulted in sharper delineation of vascular structures and an improvement in signal:noise. When image averaging was not possible the single best OCTA image was used for analysis as shown in **B**. Note that that there are significant similarities in the appearance of vascular patterns between the Optovue (image averaged) and Heidelberg (single image) scans.

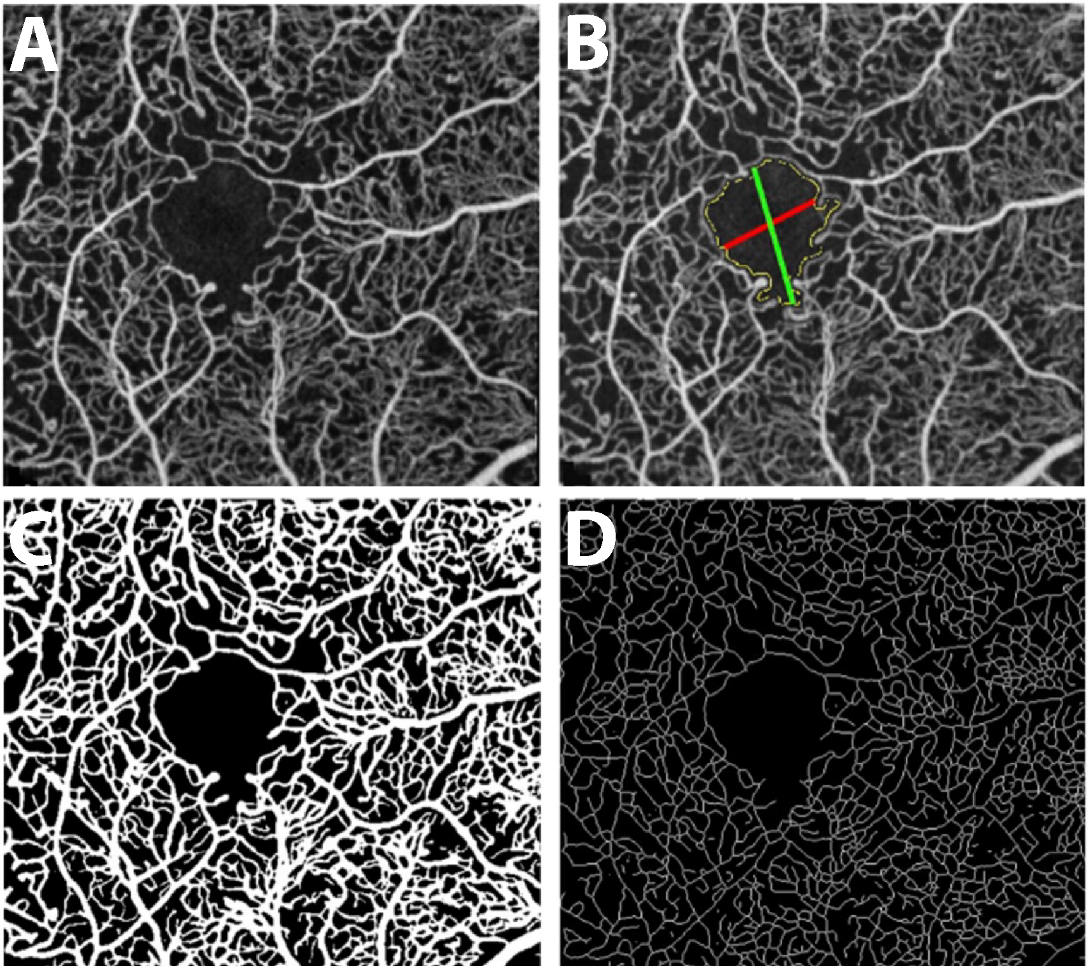

Supplementary Figure 7. Illustration of how quantifiable metrics are extracted from Optical Coherence Tomography Angiography (OCTA) images (Optovue XR Avanti). **A**, Original OCTA. **B**, Original OCTA overlaid with foveal avascular zone short axis (red), long axis (green), and perimeter (yellow) dimensions. **C**, Perfusion density derived from deep neural network vessel segmentation, where perfusion density equals the ratio of vessel pixels to total pixels. **D**, Vascular density derived as the skeletonized version of the perfusion density image, where vascular density equals the ratio of skeletonized vessel pixels to total pixels.
